# Immunohistochemical Surrogates for Molecularly Defined Renal Tumors

**DOI:** 10.3390/diagnostics16162605

**Published:** 2026-08-17

**Authors:** Roberta Mazzucchelli, Magda Zanelli, Maurizio Zizzo, Andrea Palicelli, Francesca Sanguedolce

**Affiliations:** 1 Section of Pathological Anatomy, Department of Biomedical Sciences and Public Health, United Hospitals, Università Politecnica delle Marche, 60126 Ancona, Italy; r.mazzucchelli@univpm.it; 2 Pathology Unit, Azienda USL-IRCCS di Reggio Emilia, 42123 Reggio Emilia, Italy; magda.zanelli@ausl.re.it (M.Z.); andrea.palicelli@ausl.re.it (A.P.); 3 Surgical Oncology Unit, Azienda USL-IRCCS di Reggio Emilia, 42123 Reggio Emilia, Italy; maurizio.zizzo@ausl.re.it; 4 Pathology Unit, Policlinico Foggia, University of Foggia, 71122 Foggia, Italy

**Keywords:** immunohistochemistry, biomarkers, molecularly defined renal tumors

## Abstract

The recent introduction of “molecularly defined renal cell carcinomas” in the World Health Organization classification has significantly expanded the diagnostic spectrum of renal neoplasia, highlighting entities characterized by specific genetic alterations with potential clinical and therapeutic relevance. However, the recognition of these tumors in routine practice remains challenging due to overlapping morphological features and variable access to molecular testing. In this context, immunohistochemistry (IHC) has emerged as a practical and widely available tool that can act as a surrogate for underlying molecular alterations. Depending on the biological context, IHC may reflect genetic events either through protein overexpression, as in fusion-driven tumors, or through loss of expression associated with gene inactivation in metabolically or chromatin remodeling-deficient neoplasms. Accordingly, IHC plays a central role as a screening and triage method within the diagnostic workflow of these entities. Overall, IHC remains an indispensable component in the evaluation of molecularly defined renal cell carcinomas, but its optimal use requires integration with morphological assessment and, in most cases, confirmatory molecular testing. The aim of this review is to provide a comprehensive and evidence-based overview of the main immunohistochemical surrogates used in molecularly defined renal cell carcinomas, including TFE3-, TFEB-, and ALK-rearranged tumors, as well as SDH-, FH-, and SMARCB1-deficient neoplasms, highlighting their diagnostic applications, strengths, pitfalls, and role within an integrated diagnostic workflow. For each marker, the biological rationale, expected staining patterns, diagnostic applications, and major pitfalls are discussed, with particular emphasis on variability across studies and technical limitations.

## 1. Introduction

Renal cell carcinoma (RCC) encompasses a heterogeneous group of neoplasms with distinct morphological, immunophenotypic, and molecular features. The most recent World Health Organization (WHO) classification has introduced the category of “molecularly defined renal cell carcinomas”, which includes entities characterized by specific genetic alterations that drive tumor development and, in some cases, have direct clinical and therapeutic implications [1]. These tumors often exhibit overlapping morphological features with more common RCC subtypes, posing significant diagnostic challenges in routine practice [1,2,3,4].

The category of molecularly defined renal cell carcinomas (MDRCs), introduced in the 2022 WHO classification, comprises approximately 5% of all renal cell carcinomas (RCCs) and encompasses a heterogeneous group of neoplasms defined by recurrent genetic alterations rather than morphology alone. Although collectively uncommon, these entities display strikingly diverse epidemiological profiles that often reflect their underlying molecular pathogenesis. Among them, TFE3-rearranged RCC is the most prevalent subtype, accounting for approximately 1.6–4% of adult RCCs and representing up to 40–50% of pediatric RCCs, with a mean age at diagnosis of approximately 37 years, a slight female predominance, and prior exposure to cytotoxic chemotherapy as the only established risk factor [1,2,3,4,5,6,7,8]. In contrast, TFEB-altered RCCs are considerably rarer; TFEB-rearranged tumors predominantly occur in children and young adults, whereas TFEB-amplified RCC constitutes a distinct aggressive subtype that primarily affects older adults. Metabolically driven tumors are even less frequent. SDH-deficient RCC accounts for only 0.05–0.2% of RCCs and typically presents in the fourth decade of life, shows a male predominance, and is strongly associated with germline SDH mutations. Similarly, FH-deficient RCC is a rare but highly aggressive neoplasm that develops at a younger age than conventional RCC, usually between the fourth and fifth decades of life. Although originally regarded as the renal manifestation of hereditary leiomyomatosis and renal cell carcinoma (HLRCC) syndrome, it is now recognized that approximately one-fifth of cases occur sporadically. ALK-rearranged RCC represents one of the rarest molecularly defined entities, accounting for less than 1% of RCCs, with a broad age distribution and a slight male predominance; a distinctive subgroup harboring *VCL::ALK* fusions preferentially affects children and young adults of African descent with sickle cell trait [1,2,3,4,5,6,7,8,9,10]. Likewise, SMARCB1-deficient renal medullary carcinoma is an exceptionally rare but highly aggressive neoplasm that predominantly affects adolescents and young adults with sickle cell trait or other hemoglobinopathies, whereas the recently recognized medullary-like RCC shares identical morphology but occurs in older patients without hemoglobinopathy and lacks the characteristic demographic predilections of classic renal medullary carcinoma. Finally, ELOC-mutated RCC is among the rarest MDRCs, representing approximately 1% of clear cell-type renal neoplasms and occurring predominantly in middle-aged to elderly men. Collectively, these observations highlight that molecularly defined RCCs are not only genetically distinct but also epidemiologically heterogeneous, with characteristic age distributions, sex predilections, hereditary associations, and ethnic backgrounds that may provide important diagnostic clues and should be integrated into the overall clinicopathological assessment [1,2,3,4,5,6,7,8,9,10,11,12,13].

The increasing recognition of these entities has paralleled the growing role of molecular diagnostics in renal tumor classification. Techniques such as fluorescence in situ hybridization (FISH) and next-generation sequencing (NGS) allow for precise identification of gene rearrangements, mutations, and metabolic pathway alterations. However, access to molecular testing remains variable across institutions, and these approaches may not always be feasible in the initial diagnostic workflow due to cost, turnaround time, or limited tissue availability [3,4].

In this context, immunohistochemistry (IHC) plays a critical role as an accessible, rapid, and widely available tool that can act as a surrogate for underlying molecular alterations [2]. In selected settings, IHC can provide a reliable proxy of genetic events, either through detection of protein overexpression resulting from gene fusions or through loss of expression associated with gene inactivation. Consequently, IHC represents a key component in the diagnostic approach to molecularly defined RCCs, particularly as a screening and triage method to identify cases requiring confirmatory molecular testing [3,4].

Nevertheless, the interpretation of IHC surrogates is not without limitations. The performance of these markers varies across tumor types and is influenced by biological heterogeneity, technical factors, and differences in antibody clones and laboratory protocols. Furthermore, certain markers may yield false-positive or false-negative results, and their diagnostic reliability may be inconsistent across studies. These limitations underscore the need for a structured and critical approach to the use of IHC in this setting [3,5].

This review aims to provide a practical and evidence-based overview of the main immunohistochemical surrogates used in molecularly defined renal carcinomas, with a focus on their diagnostic applications, strengths, and pitfalls. Particular attention is given to their role within the diagnostic workflow, including their utility in differential diagnosis and their integration with molecular testing strategies.

## 2. Methodology

This narrative review was based on a literature search conducted in PubMed, Scopus, and Google Scholar for studies published between January 2012 and June 2026, using combinations of keywords related to molecularly defined renal cell carcinomas and immunohistochemical surrogate markers. Original articles, review papers, and relevant classification and consensus documents published in English were considered. Studies were selected based on their relevance to the molecular basis, immunohistochemical profile, diagnostic performance, differential diagnosis, and practical limitations of the surrogate markers evaluated. Publications not specifically addressing renal tumors or not providing information relevant to the diagnostic use of the investigated markers were excluded. Additional relevant publications were identified through manual screening of the reference lists of selected articles. The evidence was critically appraised and synthesized to provide a practical, pathology-oriented overview of the current diagnostic role and limitations of immunohistochemical surrogate markers.

## 3. Principles for Interpreting IHC Surrogates

The interpretation of immunohistochemical surrogates in molecularly defined renal carcinomas requires a structured and context-driven approach that integrates morphological assessment, staining patterns, and molecular biology. A fundamental distinction must be made between markers representing a direct readout of the underlying molecular alteration and those acting as indirect or surrogate indicators. In some settings, such as ALK- or TFE3-rearranged RCCs, IHC detects overexpression of chimeric proteins resulting directly from gene fusions, thereby functioning as a direct proxy of the genetic event. In contrast, in metabolic pathway-deficient tumors, including FH- and SDH-deficient RCCs, immunohistochemical loss of protein expression represents a surrogate of enzyme dysfunction rather than a direct visualization of the genetic alteration [3,5,6].

The interpretation of staining patterns requires careful evaluation of expression type (loss versus overexpression), subcellular localization, and staining distribution. Loss-of-expression markers necessitate strict reliance on internal positive controls to exclude technical artifacts, whereas overexpression-based markers require assessment of staining intensity and specificity, as weak or nonspecific labeling may be misleading [7,8]. Sensitivity and specificity of immunohistochemical surrogates vary significantly across tumor types and markers, and are influenced by both biological and technical factors. Variability in antibody clones, fixation protocols, and laboratory procedures can substantially affect staining results, particularly for technically sensitive markers such as TFE3 and ALK. Consequently, interpretation should always be performed in conjunction with morphological features and within the appropriate clinical and pathological context [9,10,11].

Another critical aspect is the recognition that IHC often serves as a screening or triage tool rather than a definitive diagnostic method. While certain markers, such as SMARCB1 loss, may be considered highly robust in the appropriate setting, others demonstrate significant limitations, including false-positive and false-negative results, interobserver variability, and inconsistencies across studies [12]. Therefore, the integration of IHC into diagnostic workflows should be guided by a hierarchical approach, in which morphological suspicion prompts targeted immunohistochemical testing, followed by confirmatory molecular analysis when required. This approach is particularly relevant for entities with therapeutic or hereditary implications, where accurate classification is essential for patient management [2,3,5].

## 4. Renal Cell Carcinoma with Overexpression of Proteins Resulting from Gene Fusions

### 4.1. TFE3-Rearranged RCC

TFE3-rearranged RCC is defined by rearrangements of the *TFE3* gene located at Xp11.23, a member of the microphthalmia transcription factor family, in which gene fusions place *TFE3* under the control of strong promoters leading to overexpression of a chimeric protein detectable by IHC [5,13,14].

Immunohistochemical features. TFE3 IHC represents a direct readout of the underlying molecular alteration. Immunohistochemically, TFE3 shows positive expression with strong and diffuse nuclear staining in neoplastic cells, which is considered supportive of the diagnosis when technically optimal, whereas weak or “dirty” nuclear staining is nonspecific; interpretation requires strong labeling in tumor cells with absence of staining in non-neoplastic elements, although weak labeling may be observed in native podocytes [3,5,8,15]. This marker is primarily used to identify RCCs driven by Xp11.2 translocations and to support the differential diagnosis from morphologic mimics, including clear cell RCC, papillary RCC, clear cell papillary renal cell tumor, and epithelioid angiomyolipoma/PEComa [2,3,12,13]. In this context, additional immunohistochemical markers may provide diagnostic support and may assist in excluding common mimics, with frequent positivity for PAX8, CD10, and AMACR, as well as downstream markers such as GPNMB and Cathepsin K, while cytokeratins and EMA are often underexpressed or negative, GATA 3 and CD117 are generally negative, and CK7 and CA IX are typically negative or only focally expressed. On the contrary, diffuse CA IX or CK7 expression favors the diagnosis of clear cell or papillary RCC, respectively, that represents the main mimicker [3,5,8,13,16,17]. Study-level evidence highlights both diagnostic utility and limitations: in a retrospective analysis of 303 RCCs, 23.2% of immunohistochemically negative cases harbored genuine *TFE3* rearrangements, indicating that negative staining does not exclude the diagnosis, whereas other series report high positivity rates in confirmed cases, including 95% TFE3 positivity in a cohort analysis alongside high expression of PAX8 and CD10; additional studies demonstrate that GPNMB is positive in 70% of MiT family RCCs, including a substantial proportion of Cathepsin K-negative cases, and that TRIM63 in situ hybridization is highly sensitive even in Cathepsin K-negative tumors [5,13]. The main strengths of TFE3 IHC include its availability as an initial diagnostic tool and the strong diagnostic support provided by clean nuclear staining when technically optimal, as well as the increased sensitivity offered by downstream markers such as GPNMB and TRIM63; however, reproducibility is problematic due to lack of standardized interpretation criteria and interobserver variability [3,5,13].

Limitations and pitfalls. One of the disadvantages of TFE3 IHC include staining heterogeneity, frequent false-negative results related to low expression levels, and false-positive staining in other entities such as ALK-rearranged RCC, epithelioid angiomyolipoma/PEComa, and folliculin-mutated neoplasms, as well as technical issues related to fixation time and antibody clone used; in addition, there is marked variability across studies, with some reporting high sensitivity and others emphasizing unreliability, technical sensitivity, and both false-positive and false-negative results [3,5,9,10,13]. In particular, a multi-institutional study demonstrated weak-to-moderate TFE3 protein reactivity in 4 of 8 ALK-rearranged RCCs, confirming a relevant diagnostic pitfall [9,11]. Therefore, although TFE3 IHC is useful as a screening and triage tool, it is not sufficient for definitive diagnosis. Its technical limitations, susceptibility to both false-negative and false-positive results, and inter-study variability necessitate systematic molecular validation for accurate classification. Molecular confirmation is mandatory, typically by fluorescence in situ hybridization or RNA sequencing, the latter being particularly important for detecting cryptic intrachromosomal rearrangements not identifiable by FISH [2,3,8,10,13,16].

### 4.2. TFEB-Altered RCC

Transcription factor EB (TFEB)-altered RCC is characterized by alterations involving the *TFEB* gene at 6p21 and includes both TFEB-rearranged and TFEB-amplified neoplasms. In rearranged tumors, gene fusions typically involve the untranslated *MALAT1* gene, whose strong promoter drives overexpression of native TFEB protein, whereas high-level amplification similarly results in protein overexpression [3,5,13,18]. In this setting, TFEB IHC represents a direct readout of the underlying molecular event. The different alterations in *TFEB* gene affect the tumor morphology; in cases with *TFEB* rearrangement, the tumor shows a biphasic appearance composed of larger epithelioid clear-to-eosinophilic cells admixed with smaller cells clustered around eosinophilic basement membrane-like spheres, whereas TFEB-amplified tumors, that occur predominantly in older adults, display high-grade pleomorphic morphology with papillary, cribriform, or rhabdoid features [3,5,8,16,17].

Immunohistochemical features. TFEB IHC is primarily used as a screening tool to identify RCCs driven by *TFEB* rearrangement or amplification. These tumors show positive expression with strong and diffuse nuclear staining, and strong nuclear labeling is considered supportive of the diagnosis when technically validated [3,5,17]. TFEB IHC is primarily used as a screening tool to identify RCCs driven by TFEB rearrangement or amplification [2,3,5,8,16,17,19]. In this setting, ancillary markers may provide valuable support: Cathepsin K is positive in more than 90% of rearranged cases and approximately 50% of amplified tumors, Melan-A is usually diffuse and strong, HMB45 is characteristically focal but crisp, GPNMB shows high sensitivity with positivity exceeding 70%, CK20 is frequently positive in amplified tumors, and PAX8 is usually retained, whereas cytokeratins, EMA, and CA IX are typically negative or only focally expressed [2,3,5,8,17,20]. Study-level evidence further supports the diagnostic and biological relevance of these markers: a clinicopathologic and molecular study of 13 TFEB-rearranged tumors demonstrated constant PD-L1 expression in cases harboring *MALAT1*, *ACTB*, and *NEAT1* fusions, while multi-institutional and institutional studies of TFEB-amplified tumors highlighted their high-grade morphology, variable Cathepsin K and melanocytic marker expression, and frequent PD-L1 positivity; additional biomarker studies in MiT family RCCs reported 70% GPNMB positivity using a 90% threshold and high sensitivity and specificity of TRIM63 in situ hybridization compared with Cathepsin K [21,22,23]. The main strengths of TFEB-associated IHC include effective triage of tumors with biphasic or unusual high-grade morphology and the availability of sensitive downstream markers such as GPNMB and TRIM63, which may capture cases in which direct TFEB IHC or FISH is equivocal [2,3,5].

Limitations and pitfalls. Currently available TFEB antibodies are considered problematic and technically variable, and nuclear labeling may also occur in non-rearranged tumors when protocols are not strictly optimized. In addition, marked morphologic overlap with clear cell RCC and oncocytic neoplasms, false-positive interpretation in TSC/MTOR- or FLCN-mutated tumors that activate the TFEB pathway and express downstream markers, significant overlap with epithelioid angiomyolipoma, and false-negative FISH results in paracentric inversions such as *PPP1R10::TFEB*, require RNA sequencing [1,2,3,5,8,13,16].

Accordingly, although TFEB IHC is useful as a screening and triage tool, both in rearranged tumors to amplified tumors, two morphologically and biologically distinct groups, definitive diagnosis requires molecular confirmation by FISH or RNA sequencing, particularly to distinguish these tumors from mimics and to identify cryptic rearrangements not detectable by break-apart assays [3,5,17].

### 4.3. ALK-Rearranged RCC

Anaplastic lymphoma kinase (ALK) rearrangements involving the gene located on chromosome 2p23 result in the formation of oncogenic chimeric proteins that activate downstream signaling pathways, including *JAK3/STAT3*, *RAS/MEK/ERK*, and *PI3K/AKT* [9,10,21,24,25,26]. In this context, ALK IHC represents a direct readout of the underlying molecular alteration, as fusion events lead to overexpression of the abnormal ALK protein detectable by IHC [9,10,24,25].

Immunohistochemical features. ALK-rearranged RCCs typically show strong and diffuse cytoplasmic and/or membranous staining in neoplastic cells, with patterns that may vary depending on the fusion partner, including cytoplasmic–nuclear or perinuclear–cytoplasmic distributions. Diagnostic accuracy is highly dependent on the antibody clone used, with newer clones such as D5F3 and 5A4 providing higher sensitivity and specificity compared to older clones such as ALK1 [2,3,9,10,24,25,26]. This marker is primarily used to identify RCCs harboring *ALK* rearrangements and to support the differential diagnosis from a broad spectrum of morphologic mimics, including renal medullary carcinoma, collecting duct carcinoma, papillary RCC, mucinous tubular and spindle cell carcinoma, metanephric adenoma, and TFE3-rearranged RCC [2,3,9,10,12,14,15,19,25]. In this setting, additional immunohistochemical markers provide complementary information, with consistent PAX8 positivity and frequent expression of vimentin, CK7, and AMACR, while CA IX, CD117, CK20, and melanocytic markers are typically negative; retained expression of INI1/SMARCB1, FH, and SDHB is characteristic, and important diagnostic pitfalls include weak-to-moderate TFE3 immunoreactivity without gene rearrangement and TTF1 positivity in specific fusion contexts [2,3,8,9,10,16,19,25]. Not all study-level evidence supports the diagnostic utility of ALK IHC: in a multi-institutional retrospective study, all 12 cases showed diffuse ALK and PAX8 positivity, supporting its utility in screening unclassifiable RCCs, whereas, another multi-institutional study also demonstrated TFE3 protein reactivity in 4 of 8 cases without corresponding gene rearrangement, underscoring a significant diagnostic pitfall [9,10,24,25]. The main strengths of ALK IHC include its availability as a widely accessible screening tool and its clinical relevance, as identification of ALK-rearranged tumors has therapeutic implications due to potential responsiveness to targeted ALK inhibitors, with reproducible performance reported when modern antibody clones are employed [24].

Limitations and pitfalls. ALK-rearranged RCC diagnosis shows several challenges including marked morphological heterogeneity that may lead to misclassification, false-positive results related to TFE3 or TTF1 expression and alternative sources of ALK protein expression, and false-negative results associated with less sensitive antibody clones or technical factors; in addition, fluorescence in situ hybridization may fail in cases with intrachromosomal inversions, and variability across studies reflects differences in fusion partners and technical approaches [2,3,9,10,11,25,26]. Accordingly, although strong ALK expression by IHC provides a reliable initial surrogate of the underlying genetic alteration and represents a useful screening and triage tool, it is not sufficient for definitive diagnosis, and molecular confirmation is considered mandatory to avoid misclassification due to technical variability and to guide potential targeted therapy [9,16,24].

The main features of the above-mentioned tumors are summarized in Table 1.

## 5. Renal Cell Carcinoma with Metabolic Pathway-Deficient Tumors

### 5.1. SDH-Deficient RCC

Succinate dehydrogenase (SDH)-deficient RCC is driven by biallelic inactivation, most commonly germline, of one of the genes encoding the SDH complex (*SDHA*, *SDHB*, *SDHC*, or *SDHD*), a mitochondrial enzyme complex essential for cellular metabolism [6,10,14,16,27]. In this setting, immunohistochemical loss of SDHB protein expression serves as a surrogate marker of SDH complex dysfunction irrespective of the specific mutated subunit [6,10,14,16,27].

Immunohistochemical features. SDH-deficient tumors show complete loss of cytoplasmic granular staining in neoplastic cells, reflecting loss of mitochondrial expression, and diagnosis requires complete and diffuse absence of staining in tumor cells with retained internal positive controls in non-neoplastic elements such as vascular endothelium, renal tubules, or inflammatory cells; weak or patchy expression is not diagnostic and interpretation is highly dependent on technically adequate controls [3,6,13,14,16,19,27]. SDHB IHC is primarily used to identify RCCs associated with SDH deficiency and to triage patients for hereditary syndrome screening, and it plays a key role in the differential diagnosis from eosinophilic/oncocytic mimics including renal oncocytoma, chromophobe RCC, low-grade oncocytic tumor, and FH-deficient RCC, with retained SDHB expression effectively excluding the diagnosis [3,6,16,27]. In this context, additional markers may assist but are not specific, including consistent PAX8 positivity, frequent expression of GATA3 and L1CAM, and variable EMA expression, while CK7, CD117, TFE3, HMB45, Melan-A, and AMACR are typically negative; however, GATA3 and L1CAM positivity may overlap with low-grade oncocytic tumor and represent a diagnostic pitfall, reinforcing the central role of SDHB IHC [2,3,13,16,19,27]. Study-level evidence supports the robustness of SDHB loss as a defining feature: in an original series of 36 cases, SDHB loss was established as the hallmark surrogate for the entity, and in a multi-institutional study of 40 cases, 100% demonstrated SDHB loss, with frequent co-expression of GATA3 (95%) and L1CAM (72%), highlighting both sensitivity and lack of specificity of ancillary markers; a national survey further showed that diagnostic recognition is limited, with only 42.9% of centers encountering at least one case, and that morphology and patient age are the primary triggers for testing [6,27]. The main strengths of SDHB IHC include its role as a universal surrogate marker due to instability of the SDH complex following mutation of any subunit and its high reproducibility in prototypical cases with characteristic cytoplasmic vacuolation [6,10,16,27].

Limitations and pitfalls. However, several issues must be considered, including morphological heterogeneity with occasional high-grade or variant patterns, false-positive interpretation of loss in tumors with low mitochondrial content such as clear cell RCC, and false-negative or misleading interpretations in cases with faint or decreased staining if internal controls are not rigorously assessed; in addition, overlap with entities such as low-grade oncocytic tumor and limited availability of testing in non-specialized centers represent further challenges [2,3,6,8,13,19,25,27]. Although earlier studies emphasized an indolent phenotype, more recent data highlight an expanded morphological and clinical spectrum including high-grade and sarcomatoid variants, indicating partial variability across studies [2,3,6,13,16,19,27]. Accordingly, while SDHB IHC represents a first-line and decisive screening tool, molecular confirmation is required to establish germline status and is recommended in equivocal cases [3,6,16,27]. Overall, SDH-deficient RCC is a rare molecularly defined neoplasm characterized by bland oncocytic cells with distinctive cytoplasmic vacuolation, and complete loss of SDHB expression represents the mandatory diagnostic surrogate for SDH complex inactivation, with important implications for identification of hereditary paraganglioma–pheochromocytoma syndromes and appropriate genetic counseling [2,3,6,10,13,16,17].

### 5.2. FH-Deficient RCC

Fumarate hydratase (FH), also referred to as fumarase, is encoded by a gene located on chromosome 1q42.3–q43 and functions as a key enzyme in the tricarboxylic acid cycle catalyzing the conversion of fumarate to L-malate; biallelic inactivation of this gene, either germline or somatic, underlies FH-deficient RCC [3,8,13,15,17]. In this context, loss of FH protein expression by IHC represents a surrogate marker of the underlying genetic alteration [6,18].

Immunohistochemical features. FH-deficient tumors characteristically show loss of cytoplasmic staining in neoplastic cells, with complete absence of expression being typical, although retained or intact staining may occur in rare cases, particularly in the setting of missense mutations; correct interpretation requires the presence of intact internal positive controls in non-neoplastic elements such as vascular endothelium, renal tubules, or inflammatory cells [3,6,7,8,10,13,14,17,28]. Loss of FH marker is primarily used for the screening and identification of FH-deficient RCC and plays a key role in the differential diagnosis from high-grade papillary RCC, collecting duct carcinoma, tubulocystic RCC, and other high-grade infiltrative neoplasms [2,3,6,8,12,13,19,21]. In this diagnostic setting, additional immunohistochemical markers provide important complementary information, most notably 2-succinocysteine (2SC), which shows diffuse and strong nuclear and cytoplasmic positivity as a surrogate of fumarate accumulation, as well as PAX8 and CD10, which are frequently positive, while CK7 is usually negative or only focally expressed, and TFE3, HMB45/Melan-A, and CD117 are typically negative; GATA3 may be positive in a subset and represent a potential diagnostic pitfall [3,7,8,13,16,17,19,28]. Study-level evidence supports the diagnostic performance of FH and 2SC markers and in a biomarker evaluation study, 19 of 20 cases (95%) showed FH loss and all cases (20/20, 100%) were 2SC positive, indicating high sensitivity of the combined panel, whereas in a separate clinicopathologic series the sensitivity of FH IHC was approximately 87%, and not all germline-mutated cases showed loss of expression; additionally, a specificity study demonstrated 2SC positivity in 14 of 122 non–FH-deficient tumors (11.5%), predominantly oncocytic neoplasms, indicating imperfect specificity [7]. The main strengths of FH IHC include its wide availability, cost-effectiveness, and ability to provide a rapid surrogate for molecular alterations, particularly when combined with highly sensitive markers such as 2SC, with reproducible performance in typical cases when morphology and internal controls are appropriately assessed [2,7,25,28].

Limitations and pitfalls. However, several limitations must be considered, including marked morphological heterogeneity leading to frequent overlap with other high-grade RCC subtypes, false-negative results due to retained or patchy FH expression in certain mutations, and false-positive 2SC staining in a subset of non–FH-deficient tumors, as well as technical issues related to antibody availability and fixation protocols as suboptimal processing may mimic loss of expression. In addition, variability in reported sensitivity, ranging from approximately 80% to 87%, underscores inconsistencies across studies [3,6,7,13,15,16,18,19,21]. Accordingly, although the combined demonstration of FH loss and 2SC positivity provides a strong surrogate of the underlying molecular alteration, it is not sufficient for definitive diagnosis, and molecular confirmation is mandatory to establish the diagnosis and to determine germline status [3,13,16,18]. In fact, are well known cases with retained FH expression in specific mutational contexts with imperfect specificity for 2SC, that necessitate careful interpretation and mandatory molecular validation, with important implications for genetic counseling and identification of hereditary leiomyomatosis and RCC syndrome [3,7,8,13,15,16,17,19].

### 5.3. SMARCB1-Deficient Renal Medullary Carcinoma

SMARCB1, also known as INI1, hSNF5, or BAF47, is encoded by a gene located on chromosome 22q11.23 and functions as a core subunit of the SWI/SNF chromatin remodeling complex; biallelic inactivation of this gene through mutations or deletions underlies SMARCB1-deficient renal medullary carcinoma and related medullary-like RCCs [12,13,16,28,29]. In this setting, immunohistochemical loss of SMARCB1/INI1 protein expression serves as a surrogate marker for the underlying genetic alteration [2,8,28].

Immunohistochemical features. SMARCB1-deficient RCCs show complete loss of nuclear staining in neoplastic cells for SMARCB1 marker, with intact nuclear expression in internal positive controls such as adjacent immune cells, vascular endothelium, or normal renal tubules, which is essential for correct interpretation [2,3,8,12,13,14,28,29]. Loss of SMARCB1 expression is a requisite diagnostic feature in the appropriate morphological and clinical context and is central to distinguishing renal medullary carcinoma from mimics such as collecting duct carcinoma, high-grade urothelial carcinoma, ALK-rearranged RCC, and FH-deficient RCC, which typically retain SMARCB1 expression [2,3,8,10,12,16,28,30]. In this differential setting, additional immunohistochemical markers may assist, including consistent PAX8 positivity and expression of pancytokeratin and EMA, while OCT3/4 may be positive in a subset of cases and represent a diagnostic pitfall by mimicking germ cell tumors; conversely, GATA3 and p63 are typically negative, and retained SDHB and FH expression helps exclude metabolic mimics [2,3,12,13,19,28,29]. Study-level evidence supports the robustness of this marker: retrospective and cohort analyses consistently report complete nuclear loss of SMARCB1 in all included cases, including series of 54 SMARCB1-deficient neoplasms and 18 medullary or medullary-like tumors, while large cohort data required loss of SMARCB1 expression for inclusion and identified distinct clinicopathologic subsets; molecular studies further confirmed biallelic inactivation in 84.2% of cases, supporting immunohistochemical loss as a surrogate for complex genetic events, and OCT3/4 positivity has been reported in 39% to 71% of cases, underscoring its potential as both a marker and a pitfall [12,29,30,31,32]. The main strengths of SMARCB1 IHC include its widespread availability, and the complete loss of SMARCB1 nuclear expression represents essential diagnostic hallmark for SMARCB1-deficient renal medullary carcinoma. It can be considered as a robust and reproducible surrogate for its defining biallelic gene inactivation, allowing the diagnosis even in the absence of hemoglobinopathy [2,8,10,12,13,16,25,30].

Limitations and pitfalls. However, for the SMARCB1 marker, some drawbacks must be considered such as marked architectural heterogeneity that overlaps with other high-grade renal neoplasms, false-positive interpretation in cases of secondary SMARCB1 loss in other tumor types, and false-negative or equivocal results related to antibody performance or weak antigen preservation resulting in patchy staining. Additionally, approximately 10–15% of cases with immunohistochemical loss lack identifiable genetic mutations, suggesting alternative inactivation mechanisms, and diagnostic confusion may arise with morphologic mimics such as collecting duct carcinoma, urothelial carcinoma, ALK-rearranged RCC, or pediatric malignant rhabdoid tumor [2,3,5,6,8,12,14,28,29]. Accordingly, although SMARCB1 loss by IHC represents a highly reliable and often sufficient diagnostic surrogate in the appropriate context, molecular confirmation may be required in equivocal cases or to distinguish true SMARCB1-deficient renal medullary carcinoma from tumors with secondary loss [2,3,5,6,8,12,14,28,29].

The main features of the above-mentioned tumors are summarized in Table 2.

## 6. Future Perspective

The evolving landscape of molecularly defined RCCs is likely to further expand the role of immunohistochemical surrogates, particularly as diagnostic frameworks increasingly integrate morphological, genomic and proteomic data. Advances in tumor biology are likely to uncover additional protein-level correlates of genomic alterations, particularly in metabolically driven neoplasms and chromatin remodeling-deficient tumors. In this context, the future of IHC will not be limited to single-marker interpretation, but will instead move toward more integrated and quantitative approaches.

One of the most promising tools is the multiplex IHC, which allow simultaneous evaluation of multiple biomarkers within the same tissue section. These technologies have the potential to better capture tumor heterogeneity and to provide a more nuanced representation of pathway activation and tumor microenvironment interactions, thereby overcoming some of the intrinsic limitations of single-marker assays [33].

In parallel, the integration of digital pathology and artificial intelligence-based image analysis is expected to significantly improve the reproducibility and objectivity of immunohistochemical interpretation. Automated quantification of staining intensity, pattern recognition, and the development of predictive algorithms may help reduce interobserver variability and refine diagnostic thresholds, particularly for markers with known interpretative challenges. Furthermore, the combination of digital image analysis with molecular and clinical data may enable the development of integrated diagnostic models capable of predicting underlying genetic alterations from histological and immunophenotypic features [34].

## 7. Conclusions

Molecularly defined renal cell carcinomas have expanded the diagnostic spectrum of renal neoplasia and highlighted the need for an integrated approach combining morphology, immunohistochemistry, and molecular testing. Within this framework, immunohistochemistry represents an essential and widely available tool for the identification and initial characterization of these rare entities, serving primarily as a screening and triage method to guide subsequent molecular investigations.

However, the diagnostic performance of immunohistochemical surrogate markers varies considerably according to the underlying molecular alteration and should always be interpreted in the appropriate morphological and clinical context. Rather than replacing molecular testing, immunohistochemistry should be viewed as a complementary approach that improves diagnostic efficiency and optimizes the selection of cases requiring confirmatory molecular analyses. Continued advances in immunohistochemical biomarkers and molecular diagnostics are expected to further refine the classification and diagnostic work-up of these tumors.

## Figures and Tables

**Table 1:** Renal cell carcinoma with overexpression of proteins resulting from gene fusions.

	Differential Diagnosis	Molecular Alteration	Methods of Molecular Assessment	Surrogate IHC	Other IHC
**TFE3-rearranged** **RCC**	Clear cell RCC, papillary RCC, clear cell papillary renal cell tumor, and epithelioid angiomyolipoma/PEComa	Rearrangement of the TFE3 gene located at Xp11.23 chromosome	Break-apart FISH or RNA sequencing	TFE3 nuclear staining in neoplastic cells	**Negativity for**: CK7, CA IX, GATA3, CD117 **Positivity for:** PAX8, CD10, AMACR, GPNMB, Cathepsin K
**TFEB-altered RCC**	Clear cell RCC, clear cell papillary renal cell tumor, oncocytoma, and epithelioid angiomyolipoma	Rearrangement and amplification of TFEB gene at 6p21 chromosome.	Break-apart FISH or RNA sequencing	TFEB strong and diffuse nuclear staining in neoplastic cells	**Negativity for:** Cytokeratins, EMA, CA IX **Positivity for:** PAX8, CK20, GPNMB, Cathepsin K, Melan-A, HMB45 (focal)
**ALK-rearranged RCC**	Renal medullary carcinoma, collecting duct carcinoma, papillary RCC, mucinous tubular and spindle cell carcinoma, metanephric adenoma, and TFE3-rearranged RCC	Rearrangement of ALK gene located at 2p23 chromosome	Break-apart FISH or sequencing	ALK strong and diffuse cytoplasmic and/or membranous staining	**Negativity for:** CA IX, CD117, CK20, melanocytic markers **Positivity for:** PAX8, vimentin, CK7, AMACR INI1/SMARCB1, FH, SDHB (retained)

**Table 2:** Renal cell carcinoma with metabolic pathway-deficient tumors.

	Differential Diagnosis	Molecular Alteration	Methods of Molecular Assessment	Surrogate IHC	Other IHC
**SDH-deficient RCC**	Eosinophilic/oncocytic mimics including renal oncocytoma, chromophobe RCC, low-grade oncocytic tumor, and FH-deficient RCC	Biallelic inactivation of one of the genes encoding the SDH complex a mitochondrial enzyme complex essential for cellular metabolism	Next-generation sequencing and DNA sequencing	SDHB complete loss of cytoplasmic granular staining in neoplastic cells with internal intact controls	**Negativity for:** CK7, CD117, TFE3, HMB45, Melan-A, AMACR **Positivity for:** GATA3, L1CAM
**FH-deficient RCC**	High-grade papillary RCC, collecting duct carcinoma, tubulocystic RCC	Biallelic inactivation of a gene that functions as a key enzyme in the tricarboxylic acid cycle catalyzing the conversion of fumarate to L-malate	Next-generation sequencing and DNA sequencing	FH loss of cytoplasmic staining in neoplastic cells staining with intact internal positive controls 2-succinocysteine (2SC), which shows diffuse and strong nuclear and cytoplasmic positivity as a surrogate of fumarate accumulation	**Negative for:** CK7, TFE3, HMB45/Melan-A, CD117 **Positivity for:** PAX8, CD10, GATA3 (may be positive), INI1/SMARCB1 (retained)
**SMARCB1-deficient Renal Medullary Carcinoma**	Collecting duct carcinoma, high-grade urothelial carcinoma, ALK-rearranged RCC, and FH-deficient RCC	Biallelic inactivation of gene that functions as a core subunit of the SWI/SNF chromatin remodeling complex	FISH and mutational analysis	SMARCB1 (INI1) complete loss of nuclear staining with intact nuclear expression in internal positive controls	**Negativity for:** GATA3 and p63 **Positivity for:** PAX8, pancytokeratin, EMA, OCT3/4 (may be positive)

## Data Availability

No new data were created or analyzed in this study. Data sharing is not applicable to this article.

## References

[1] WHO Classification of Tumours Editorial Board (2022). WHO Classification of Tumours, Urinary and Male Genital Tumours.

[2] Orsatti A., Fernandes-Pontes F., Silva J.R.E., Tavares N.T., Jerónimo C., Henrique R., Rodrigues Â., Ricci C., Lobo J. (2026). Advances in Renal Cell Carcinoma Diagnosis: A Review on Biomarkers. Pathobiology.

[3] Akgul M., George R.S., Siegmund S.E., Oktay M., Sangoi A.R., Williamson S.R., Cheng L. (2026). Molecularly defined renal cell carcinomas: Practical approaches for surgical pathologists. Histopathology.

[4] Akbulut D., Sangoi A.R., Williamson S.R., Akgul M. (2024). Are Renal Tumor Diagnostics Becoming Too Advanced for Many Pathology Laboratories?. Int. J. Surg. Pathol..

[5] Argani P. (2026). MiT family translocation carcinomas of the kidney and related entities. Histopathology.

[6] Fanelli G.N., Caliò A., Marletta S., Ballotta M., Barella M., Bellezza G., Bianco P., Bonadio A.G., Colombo P., D’Amuri A. (2026). Diagnostic practice and awareness of SDH- and FH-deficient renal cell carcinoma: Results from an Italian Study Group of uropathology (GIUP) survey. Virch. Arch..

[7] Mannan R., Wang X., Bawa P.S., Chugh S., Chinnaiyan A.K., Rangaswamy R., Zhang Y., Cao X., Smith S.C., Trpkov K. (2023). Characterization of protein S-(2-succino)-cysteine (2SC) succination as a biomarker for fumarate hydratase-deficient renal cell carcinoma. Hum. Pathol..

[8] Raghavan A.A., Gibson I.W., Wightman R., Czaykowki P., Graham J. (2023). Redefining Renal Cell Carcinoma: A Molecular Perspective on Classification and Clinical Implications. EMJ.

[9] Zhao M., Yin X., Yang X., Gan H., Chen N., Duan G., Bai Y., Teng X., Xu J., Fang R. (2024). ALK-Rearranged Renal Cell Carcinoma: A Multi-Institutional Study of 9 Cases With Expanding the Morphologic and Molecular Genetic Spectrum. Mod. Pathol..

[10] Zhang X., Ban C., Chen Y., Zhang S., Chen H. (2025). ALK-Rearranged Renal Cell Carcinoma: A Study of Three Cases With Clinicopathologic Features and Effect of Postoperative Adjuvant Immunotherapy. Clin. Genitourin. Cancer.

[11] Lobo A., Akgul M., Sangoi A.R., Al-Obaidy K.I., Acosta A.M., Kandukuri S.R., Kapoor R., Mishra S.K., Jha S., Kaushal S. (2026). ALK-Rearranged Renal Cell Carcinoma: Morphologic Spectrum and Genomic Landscape From a Multi-Institutional Cohort of 16 Cases. Am. J. Surg. Pathol..

[12] Luo W., Zheng Y., Huang W., Jiang Y., Su B., Zhang N. (2026). Clinicopathological features of SMARCB1/INI1-deficient medullary-like renal cell carcinoma: Report of 3 cases and literature review. Ann. Diagn. Pathol..

[13] Hu X., Tan C., Zhu G. (2023). Clinical Characteristics of Molecularly Defined Renal Cell Carcinomas. Curr. Issues Mol. Biol..

[14] Bahlinger V., Lange F., Eckstein M. (2024). Molecular uropathology: What a practicing pathologist should know. Diagn. Histopathol..

[15] Mohanty S.K., Lobo A., Cheng L. (2023). The 2022 revision of the World Health Organization classification of tumors of the urinary system and male genital organs: Advances and challenges. Hum. Pathol..

[16] Kanakaraj J., Chang J., Hampton L.J., Smith S.C. (2024). The New WHO Category of “Molecularly Defined Renal Carcinomas”: Clinical and Diagnostic Features and Management Implications. Urol. Oncol..

[17] Caliò A., Marletta S., Brunelli M., Martignoni G. (2022). WHO 2022 Classification of Kidney Tumors: What is relevant? An update and future novelties for the pathologist. Pathologica.

[18] Zhang X., Bolck H.A., Rupp N.J., Moch H. (2024). Genomic alterations and diagnosis of renal cancer. Virch. Arch..

[19] Li J., Wilkerson M.L., Deng F.M., Liu H. (2024). The Application and Pitfalls of Immunohistochemical Markers in Challenging Diagnosis of Genitourinary Pathology. Arch. Pathol. Lab. Med..

[20] Saleeb R.M., Castillo V.F., Aron M., Amin M.B. (2026). The next chapter in renal cell carcinoma: Emerging entities and diagnostic refinements beyond the WHO 5th edition (2022–2025). Diagn. Histopathol..

[21] Caliò A., Brunelli M., Gobbo S., Argani P., Munari E., Netto G., Martignoni G. (2021). Cathepsin K: A Novel Diagnostic and Predictive Biomarker for Renal Tumors. Cancers.

[22] Salles D.C., Asrani K., Woo J., Vidotto T., Liu H.B., Vidal I., Matoso A., Netto G.J., Argani P., Lotan T.L. (2022). GPNMB expression identifies TSC1/2/mTOR-associated and MiT family translocation-driven renal neoplasms. J. Pathol..

[23] Kammerer-Jacquet S.F., Gandon C., Dugay F., Laguerre B., Peyronnet B., Mathieu R., Verhoest G., Bensalah K., Leroy X., Aubert S. (2022). Comprehensive study of nine novel cases of TFEB-amplified renal cell carcinoma: An aggressive tumour with frequent PDL1 expression. Histopathology.

[24] Arizpe D., Gallan A.J., Kase A.M., Krishna M., Dasari S., Tekin B., Jimenez R.E., Erickson L.A., Cheville J.C., Gupta S. (2025). ALK-Rearranged renal neoplasms: Update on an institutional cohort. Virch. Arch..

[25] Kuroda N., Trpkov K., Gao Y., Tretiakova M., Liu Y.J., Ulamec M., Takeuchi K., Agaimy A., Przybycin C., Magi-Galluzzi C. (2020). ALK rearranged renal cell carcinoma (ALK-RCC): A multi-institutional study of twelve cases with identification of novel partner genes CLIP1, KIF5B and KIAA1217. Mod. Pathol..

[26] Galea L.A., Hildebrand M.S., Witkowski T., Joy C., McEvoy C.R., Hanegbi U., Aga A. (2023). ALK-rearranged renal cell carcinoma with TPM3::ALK gene fusion and review of the literature. Virch. Arch..

[27] Sangoi A.R., Williamson S.R., Oktay M., Gill A.J., Trpkov K., Siadat F., MacLean F., Galea L.A., Baydar D.E., Cakir C. (2025). Succinate dehydrogenase deficient renal cell carcinoma frequently expresses GATA3 and L1CAM. Virch. Arch..

[28] Whaley R.D., Msaouel P., Cheville J.C., Gupta S. (2025). Renal Medullary Carcinoma: A Contemporary Update. Kidney Cancer.

[29] Sanchez S.I., Adams C., Illei P., Ali S.Z., Rooper L., Lih T.M., Li Q.K. (2025). Cytopathologic and histopathologic characteristics of SMARCB1 deficient neoplasm and correlation with molecular and immunohistochemical findings. Hum. Pathol..

[30] Lebenthal J.M., Kontoyiannis P.D., Hahn A.W., Lim Z.D., Rao P., Cheng J.P., Chan B., Daw N.C., Sheth R.A., Karam J.A. (2025). Clinical Characteristics, Management, and Outcomes of Patients with Renal Medullary Carcinoma: A Single-center Retrospective Analysis of 135 Patients. Eur. Urol. Oncol..

[31] Msaouel P., Malouf G.G., Su X., Yao H., Tripathi D.N., Soeung M., Gao J., Rao P., Coarfa C., Creighton C.J. (2020). Comprehensive Molecular Characterization Identifies Distinct Genomic and Immune Hallmarks of Renal Medullary Carcinoma. Cancer Cell.

[32] Rao P., Tannir N.M., Tamboli P. (2012). Expression of OCT3/4 in renal medullary carcinoma represents a potential diagnostic pitfall. Am. J. Surg. Pathol..

[33] Taube J.M., Sunshine J.C., Angelo M., Akturk G., Eminizer M., Engle L.L., Ferreira C.S., Gnjatic S., Green B., Greenbaum S. (2025). Society for Immunotherapy of Cancer: Updates and best practices for multiplex immunohistochemistry (IHC) and immunofluorescence (IF) image analysis and data sharing. J. Immunother. Cancer.

[34] Zhang J., Choi H., Kim Y., Park J., Cho S., Kim E., Cho A., Lee J.Y., Choi J., Low C. (2025). Artificial intelligence-based digital pathology using H&E-stained whole slide images in immuno-oncology: From immune biomarker detection to immunotherapy response prediction. J. Immunother. Cancer.

